# Potential Antivirals: Natural Products Targeting Replication Enzymes of Dengue and Chikungunya Viruses

**DOI:** 10.3390/molecules22030505

**Published:** 2017-03-22

**Authors:** Ana Flávia Costa da Silveira Oliveira, Róbson Ricardo Teixeira, André Silva de Oliveira, Ana Paula Martins de Souza, Milene Lopes da Silva, Sérgio Oliveira de Paula

**Affiliations:** 1 Departamento de Biologia Geral, Universidade Federal de Viçosa, Av. P.H. Rolfs, S/N, 36570-900 Viçosa, MG, Brazil; ana.oliveira@ifnmg.edu.br (A.F.C.d.S.O.); andre.oliveira@ifnmg.edu.br (A.S.d.O.); 2 Instituto Federal de Educação, Ciência e Tecnologia do Norte de Minas, 39900-000 Almenara, MG, Brazil; 3 Departamento de Química, Universidade Federal de Viçosa, Av. P.H. Rolfs, S/N, 36570-900 Viçosa, MG, Brazil; anapaulamartins20@hotmail.com (A.P.M.d.S.); milenelopesdasilva@yahoo.com.br (M.L.d.S.)

**Keywords:** dengue, chikungunya, virus enzymes, antiviral, natural products

## Abstract

Dengue virus (DENV) and chikungunya virus (CHIKV) are reemergent arboviruses that are transmitted by mosquitoes of the *Aedes* genus. During the last several decades, these viruses have been responsible for millions of cases of infection and thousands of deaths worldwide. Therefore, several investigations were conducted over the past few years to find antiviral compounds for the treatment of DENV and CHIKV infections. One attractive strategy is the screening of compounds that target enzymes involved in the replication of both DENV and CHIKV. In this review, we describe advances in the evaluation of natural products targeting the enzymes involved in the replication of these viruses.

## 1. Introduction

Neglected tropical diseases (NTDs) compose a group of infections that primarily affects the world’s poorest populations; they continue to be the leading cause of morbidity and mortality among the poorest people in developing countries [1,2,3,4]. NTDs affect more than one billion individuals who have inadequate access to safe water, sanitation and appropriate housing. The NTDs are caused by helminths, protozoa and tropical bacteria. Because of their link to poverty in developing countries, diseases caused by several arboviruses, including dengue virus (DENV) and chikungunya virus (CHIKV), should be considered NTDs [3].

CHIKV is a mosquito-transmitted alphavirus that belongs to the Togaviridae family [5]. It is a reemergent arbovirus that is responsible for chikungunya fever (CHIKF), severe joint pain and rash [6]. CHIKF was first described in 1952 after an outbreak along the borders of Tanzania (former Tanganyika) and Mozambique. This alphavirus is transmitted by mosquitoes of the *Aedes* genus, including *A. aegypti*, *A. furcifer* and *A. albopictus* [6,7]. In 2006, CHIKV was responsible for an epidemic of unprecedented magnitude in the Indian Ocean, stressing the need for new therapeutic approaches. Since that time, researchers have acquired a better understanding of CHIKV biology, which should lead to the identification of new, active molecules against this reemergent pathogen [8].

DENV, like CHIKV, is a reemergent arbovirus. Within the last two decades, it has become the most important and frequently transmitted arbovirus worldwide [9]. The incidence of DENV has increased approximately 30-fold over the past 50 years. Approximately 50–100 million new infections are estimated to occur annually in more than 100 endemic countries, and further spread has been documented to areas that were previously unaffected. Each year, hundreds of thousands of serious cases of infection arise, which result in approximately 20,000 deaths; additionally, 264 disability-adjusted life years per million inhabitants per year are lost, with an estimated cost for outpatient cases and hospitalizations ranging from US$514–1394. The actual numbers are probably much worse, since the underreporting of DENV cases has been documented [9,10,11,12,13]. This disease comprises four viral serotypes (DENV 1–4), and its spectrum ranges from asymptomatic infection to dengue fever, dengue hemorrhagic fever and dengue shock syndrome. Moreover, DENV infection may lead to death. All four serotypes of DENV are transmitted to humans by *A. aegypti* and *A. albopictus* mosquitoes [14,15,16].

Considering the severity of these diseases, several investigations have been conducted during the past few years to identify antiviral compounds with which to treat them. The development of antiviral therapies remains a high priority for the World Health Organization (WHO).

## 2. Natural Products as a Source of Pharmaceuticals

Several approaches have been used for drug discovery [17], including the use of natural products, which can be explored for the research and development of new pharmaceuticals. Several drugs for treating a variety of diseases have been discovered via the screening of natural compounds obtained from animals, microorganisms, marine organisms and plants. Such drugs include natural products, semi-synthetic analogs derived from active natural products and entirely synthetic compounds designed using natural products as models. Since the early days of drug discovery, chemical constituents isolated from natural sources have been explored as a source of novel therapeutics. Statistically, about 50% of new chemical entities are obtained from either natural products or natural product analogs [18,19,20,21].

Several plants have been shown to possess anti-DENV and anti-CHIKV activities. According to a WHO factsheet, 80% of the population of certain Asian and African countries depends on traditional medicines for primary healthcare due to economic and geographic constraints [16,22,23,24,25]. Because of their few (or unknown) adverse effects, the global use of medicinal plants or herbal-based medicines is steadily growing. Even though a number of plants is known for their anti-DENV and anti-CHIKV activities, few investigations have been published on the isolation (identification) and evaluation of compounds from plants with anti-DENV and anti-CHIKV activities [16].

In this review, we describe advances in the evaluation of natural products targeting the enzymes involved in the replication of DENV and CHIKV. Most of the compounds described below are natural products isolated from plant species. However, examples of substances isolated from other natural sources are also presented. Investigations of plant extracts that possess antiviral activities are also discussed. In addition, the results of in silico studies are also highlighted.

## 3. Structural Biology of DENV

DENV particles are about 50 nm in diameter. The DENV RNA genome of 10,723 nucleotides encodes an uninterrupted open reading frame, which directs the synthesis of the polyprotein precursor NH2-C-prM-E-NS1-NS2A-NS2B-NS3-NS4A-NS4B-NS5-COOH, where C is the capsid protein, M is a membrane-associated protein, E is the envelope protein and NS1-NS5 are nonstructural proteins [25,26,27].

Proteins NS3 and NS5 are integral components of the viral replication complex and are involved in viral RNA genome synthesis, methylation of the 5′ cap of the viral genome and polyprotein processing, among other activities [19].

### 3.1. NS3

NS3 is a multifunctional enzyme, which acts as a protease for polyprotein processing, an RNA triphosphatase for capping nascent viral RNA and a helicase, along with cofactor NS2B, for unwinding the double-stranded, replicative form of RNA [28]. NS3 is sufficiently conserved within the four DENV serotypes [28]. Dissecting its structural and functional domains is thus critical to improving the understanding of the flavivirus life cycle and assisting in the design of effective antiviral drugs. Furthermore, the conservation of domain structures across the Flaviviridae family suggests conservation of functions [22,26].

The NS3 protease is required for the production of mature viruses and plays a key role in maintaining infectivity. This enzyme mediates the cleavage of polyproteins into functional proteins that are required for viral propagation. The dimeric protease NS2B-NS3 is responsible for protein processing at the junctions of NS2A/NS2B, NS2B/NS3, NS3/NS4A and NS4B/NS5, as well as internal DENV sites within C, 2A, NS3 and NS4A, thus making the nonstructural NS2B-NS3 protease an ideal target for drug design against DENV infection [29,30,31].

The helicase domain of NS3 has seven structural motifs that are reminiscent of superfamily 2 helicases. It has three subdomains with significant sequence identity and structural similarity to other flavivirus helicases. Subdomains I and II are also structurally similar to the corresponding domains in the hepatitis C virus, suggesting a common mechanism of action [26,32,33,34].

The combined activities of the polynucleotide-stimulated helicase and nucleoside triphosphatase (NTPase) regions in the C-terminal domain are required for both melting secondary structures prior to the initiation of RNA synthesis and unwinding RNA duplexes; these actions occur by either separating double-stranded RNA intermediates that are formed during viral RNA synthesis or removing proteins bound to viral RNA using a translocase function. DENV with impaired helicase activity loses its ability to replicate, demonstrating the importance of the NS3 helicase domain in the viral life cycle. For this reason, inhibitors or modulators of these enzymes are of great interest as therapeutic agents [26,35,36,37].

### 3.2. NS5

NS5 is the largest (104 kDa) and most conserved protein in DENV. Specifically, residues 320–368 are strictly conserved among the flaviviruses. These residues have also been implicated in the interaction between NS5 and NS3 [38,39]. The important role of NS5 in DENV replication makes these proteins interesting targets for virus inhibition [26]. Similar to NS3, NS5 possesses two major activities, an RNA-dependent RNA polymerase (RdRp; residues 320–900) at its C-terminal end and a methyltransferase (MTase; residues 1–296) at its N-terminal end [26,40]. The NS5 MTase is a surface polyprotein that is essential for the attachment of the virus to the host cell. Thus, the ligand-bound crystal structure of the N-terminal domain of the NS5 MTase has become a crucial tool for current drug discovery efforts. The crystal structure of the RdRp shows an active site with two zinc ion-binding motifs, which are ideal targets for designing novel RdRp inhibitors [4,41,42].

The NS5 RdRp plays a vital role in the replication of viral RNA. Following viral entry and translation of the viral genome, the NS5 RdRp performs de novo RNA synthesis, generating negative-sense RNA from the positive-sense viral RNA template. The latter then serves as a template for the synthesis of more positive-sense RNA strands, which are either used for protein translation or packaged into infectious virions [43,44,45].

The flavivirus MTase catalyzes two very distinct methylation reactions, but there is only a single binding site for the methyl donor. It is likely that the RNA substrate repositions itself after the first reaction so that the second reaction can proceed [45,46].

Natural compounds that display inhibitory effects against DENV enzymes are described below. The evaluation of a compound’s activity against viral enzymes can be investigated in two ways. First, the effect of a substance can be assessed directly on the enzyme (enzymatic assay). Second, the evaluation can be carried out in replicon cell lines (which are unable to produce infectious particles, but are capable of RNA replication) using a virus strain (post-treatment assay). For the post-treatment assay, host cells are initially infected with viruses, which can attach to and enter the cell. Then, the viruses that did not attach to the host cells are removed. Finally, the compound under investigation is added. An observation of virus inhibition indicates that the compound acts on the virus replication process, which involves several enzymes. The compound is thus assumed to act on one or more viral enzymes. Molecular docking is another important approach that has been used to investigate inhibitors of viral enzymes. The results from molecular docking experiments and in vitro analyses help streamline the screening processes.

### 3.3. Direct Inhibitory Activities of Natural Compounds against DENV Enzymes

**Figure 1:**
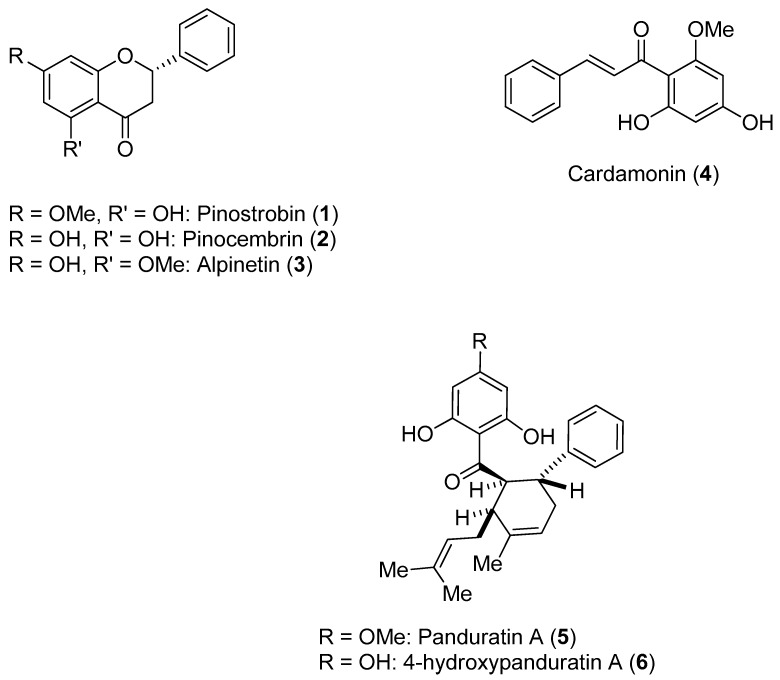
Natural products isolated from the rhizomes of *B. rotunda* (L.).

*Boesenbergia rotunda* (L.) Mansf. Kulturpfl (Br) is a member of the ginger family. A phytochemical investigation of yellow rhizomes of this species from Thailand led to the isolation of six compounds, which are shown in Figure 1.

These compounds were assayed against the DENV-2 NS3 protease [47]. The enzymatic assays were carried out using the fluorogenic peptide Boc-Gly-Arg-Arg-MCA, which is an active substrate of the DENV-2 NS3 protease. Initially, the activities of the six compounds against NS3 were evaluated at three different concentrations (120, 240 and 400 ppm); all compounds inhibited NS3 protease activity in a concentration-dependent manner. The most active compounds were **5** and **6**. The activities of these compounds were further evaluated at 40, 80 and 160 ppm. The inhibitory activity of Compound **5** ranged from 27.1% (40 ppm)–99.8% (400 ppm), whereas the inhibitory activity of Compound **6** ranged from 52.0% (40 ppm)–99.8% (400 ppm). Individually, Compounds **2** and **4** were found to have low inhibitory activity. However, when these flavones were combined, an increase in inhibitory activity was noticed (synergistic effect). Kinetic studies carried out with Compounds **1**, **4**, **5** and **6** resulted in the determination of their K_i_ values (345 for **1**; 377 for **4**; 25 for **5**; and 21 for **6**). Compounds **1** and **4** appeared to function via a noncompetitive mechanism, whereas Compounds **5** and **6** displayed competitive inhibition [47].

**Figure 2:**
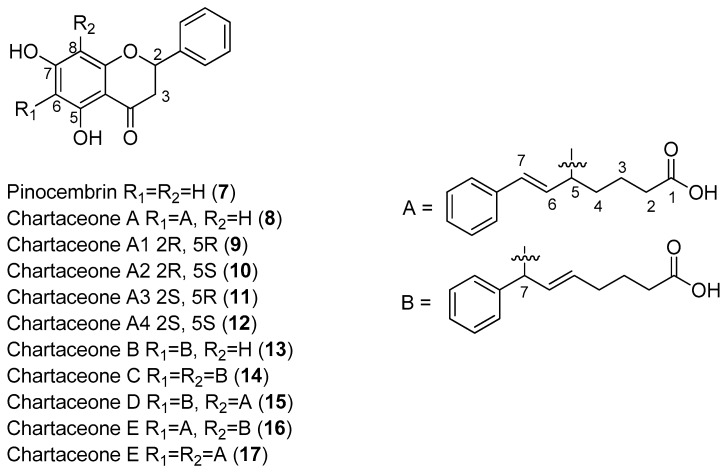
Chemical constituents isolated from the bark of *Cryptocarya chartacea*.

Litaudon and co-workers biologically screened 1350 ethyl acetate extracts obtained from various parts of 650 New Caledonian plants. The extracts were screened at a concentration of 50 μg/mL in a DENV polymerase assay using the RdRp domain of DENV-2 NS5. A second screen of 320 active extracts at a concentration of 10 μg/mL was then performed, which resulted in the selection of 49 extracts exhibiting enzymatic inhibition of at least 80%. An extract from the bark of *Cryptocarya chartacea* displayed significant enzymatic inhibitory activity (90% at 10 μg/mL) and was subsequently selected for fractionation. Flavonoids **7**–**17** (Figure 2) were isolated from this extract [48].

As can be seen in Figure 2, flavonoids **8**–**17** inhibited the DENV NS5 RdRp, with IC_50_ values ranging from 1.8 μM (Compound **15**)–72.5 μM (Compound **13**). Pinocembrin (**7**) was found to be inactive against the DENV-2 NS5 RdRp, suggesting that the aliphatic portion of the chartaceone structure plays an important role in the inhibition of the polymerase. In this investigation, the authors also evaluated the effects of the isolated compounds on nasopharyngeal carcinoma cells. No compounds were found to be toxic to this cell line at a concentration of 10 μg/mL [48].

**Figure 3:**
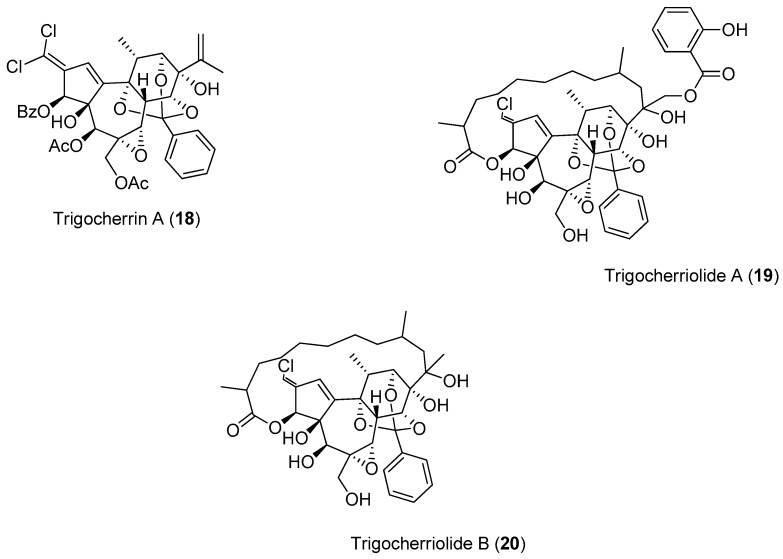
Diterpenoids from *T. cherrieri* and their antiviral effects against the dengue virus (DENV) NS5 RdRp.

The investigation of the ethyl acetate extracts of the bark and wood of *Trigonostemon cherrieri*, a rare endemic plant from New Caledonia, resulted in the isolation of a series of very complex, oxygenated diterpenoids, which included Compounds **18**–**20**. These compounds were screened against the DENV NS5 RdRp (Figure 3).

Compounds **18**–**20** were capable of inhibiting the DENV NS5 RdRp. Compound **19** was the most potent (IC_50_ = 3.1 μM), with an inhibitory activity more than 150-times lower than 3-deoxy-Guanosine Triphosphate (3-deoxy-GTP) (0.02 μM), which was used as the reference compound [49].

**Figure 4:**
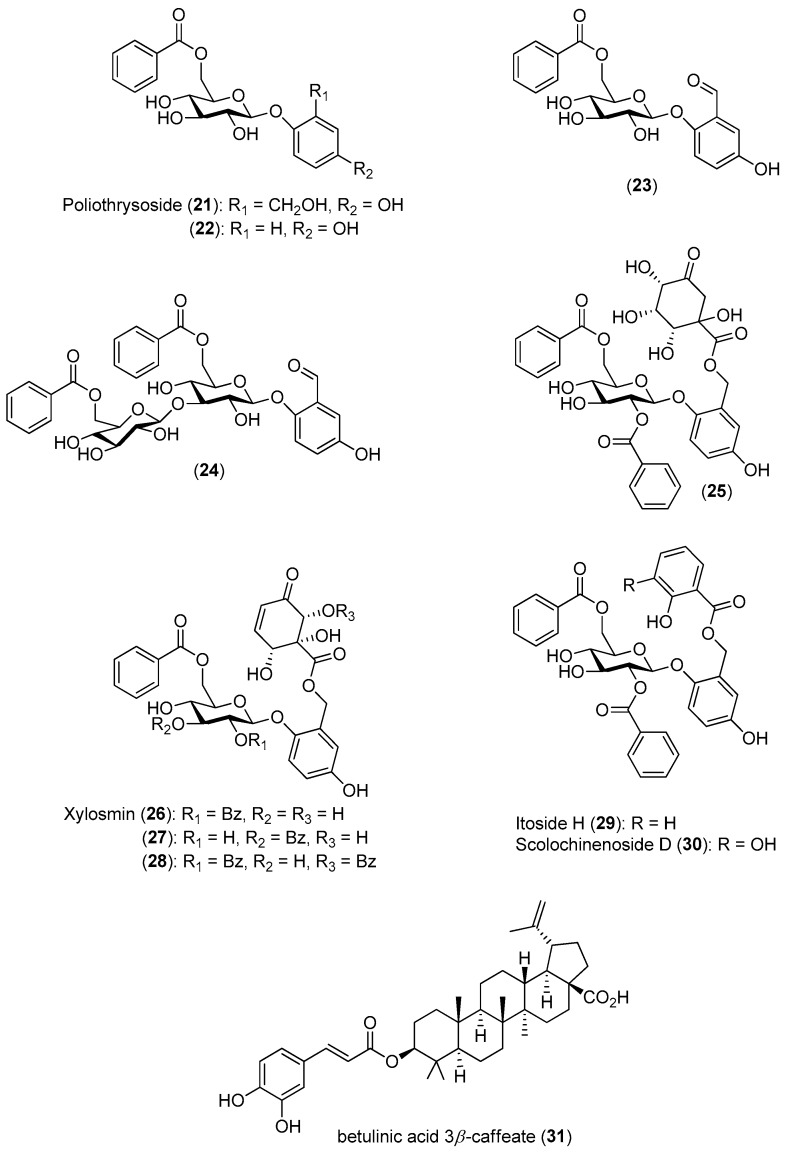
Natural compounds isolated from the ethyl acetate bark extract of *F. ramontchi* and their inhibitory effects on the DENV-2 NS5 RdRp.

To identify new inhibitors of the DENV-2 NS5 RdRp, Litaudon and co-workers screened 820 ethyl acetate extracts from different parts of 400 plants collected in Madagascar. The bark extract of *Flacourtia ramontchi* was selected for fractionation, because of its pronounced effect on the DENV-2 NS5 RdRp. The compounds shown in Figure 4 were isolated from this extract and fully characterized [50]. The data displayed in Figure 4 reveal that among the isolated phenolic glycosides **21**–**31**, Compounds **22**, **27** and **30** were the most active. The cinnamic acid derivative Compound **31** showed significant inhibitory activity, although its activity is about four-times lower than the positive control. However, no structure-activity relationships could be established for any of these compounds.

**Figure 5:**
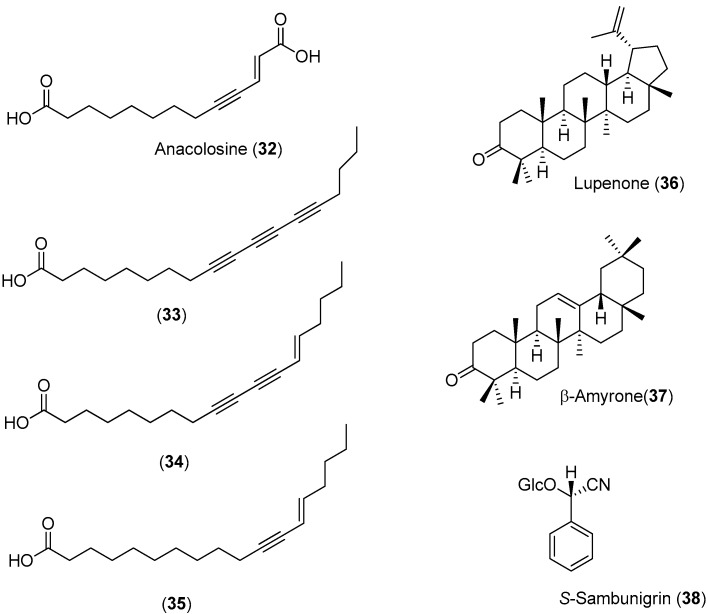
Structures of Compounds **32**–**38** and their antiviral activities.

*Anacolosa pervilleana* is a Madagascan plant whose leaves, bark and young shoots are used in traditional medicines for the treatment of schistosomiasis, syphilis and general weakness. Four acetylenic carboxylic acids (Compounds **32**–**35**), two triterpenes (Compounds **36** and **37**) and one aromatic compound (Compound **38**) were isolated from the leaf ethyl acetate extract of this species (Figure 5) [51].

The acetylenic acids were found to be the most active compounds, inhibiting the DENV NS5 RdRp with approximately the same efficacy. The observed IC_50_ values (<3 μM) were about 100-times lower than the reference compound, 3′-deoxy-GTP [51].

### 3.4. Direct Inhibitory Activities of Plant Extracts against DENV Enzymes

The medicinal plants *Vernonia cinerea*, *Hemigraphis reptans*, *Hedyotis auricularia*, *Laurentia longiflora*, *Tridax procumbens* and *Senna angustifolia* were used to evaluate their abilities to inhibit the DENV NS3 protease. The highest inhibitory effects were observed for the ethanolic extract of *S. angustifolia* leaves, the methanolic extract of *V. cinerea* leaves and the ethanolic extract of *T. procumbens* stems (IC_50_ values: 30.1 ± 3.4, 23.7 ± 4.1 and 25.6 ± 3.8 μg/mL, respectively). The most active extracts were also tested in vitro against DENV-2-infected Vero cells, which were able to maintain a normal morphology without cytopathic effects. The percent viral inhibition of the extracts of *V. cinerea* (80.6% ± 6.1%) and *T. procumbens* (64.0% ± 9.4%) was significantly higher than that of *S. angustifolia* extract (26.3% ± 3.8%), as measured by a plaque-forming assay and RT-qPCR [52]. The authors of this investigation did not analyze which compounds were responsible for the observed activities.

### 3.5. Indirect Inhibitory Activities of Compounds and Extracts against DENV Enzymes

**Figure 6:**
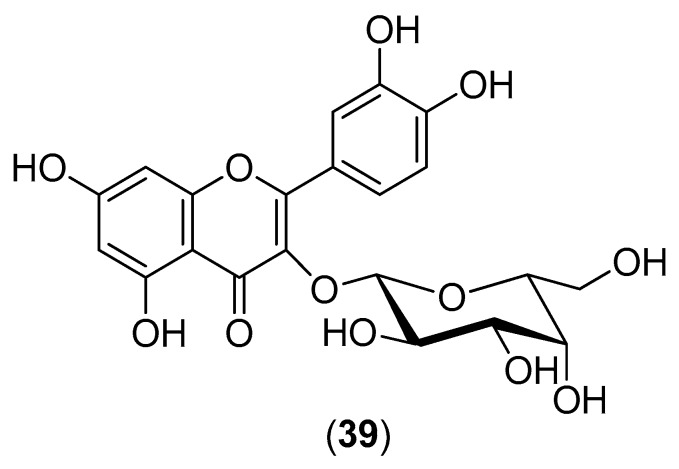
Structure of hyperoside (Compound **39**), the major component of the *H. cordata* extract.

The plant *Houttuynia cordata* is a popular vegetable consumed in the northern and eastern regions of Thailand. Its aqueous extract was evaluated for inhibitory activity against DENV-2 within the 10–100-mg/mL concentration range. Both pre- and post-incubation of HepG2 cells with *H. cordata* extract resulted in a significant reduction in intracellular DENV-2 RNA production, correlating with a decrease in DENV-2 protein expression. The extract directly inhibited intracellular viral RNA replication, with an effective concentration (EC_50_) of 0.8 mg/mL. Within the 10–40-mg/mL concentration range, the *H. cordata* extract also exhibited a protective effect on virion release from infected LLC-MK2 cells (Rhesus Monkey Kidney Epithelial Cells). The reduction in RNA production and decrease in DENV-2 protein expression suggest the inhibition of viral enzymatic activity by the aqueous extract. High-performance liquid chromatography of the aqueous extract revealed that hyperoside (Compound **39**) (Figure 6) is the major component of the extract, suggesting that this flavonoid plays an important role in DENV-2 inhibition. The aqueous extract had no toxic effects on human blood cells [53].

**Figure 7:**
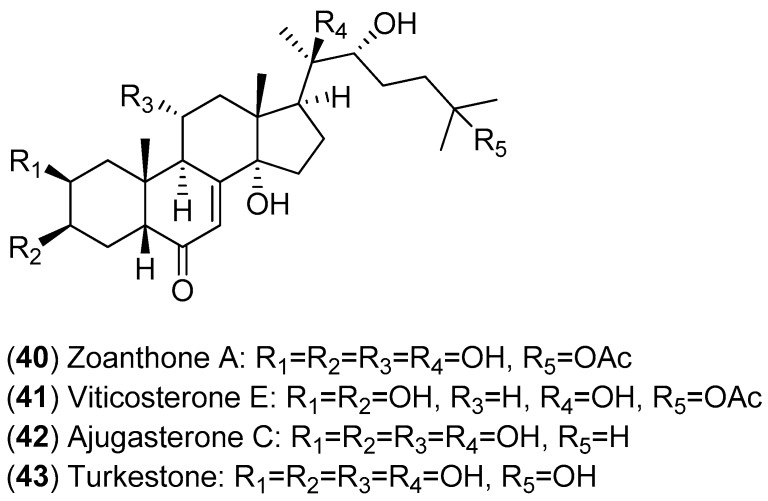
Structures of ecdysones **40**–**43**.

The fractionation of the ethanolic extract of *Zoanthus* spp. (sea anemone) collected in Taiwan resulted in the isolation of 14 ecdysones; the inhibitory activities of these compounds against DENV-2 were evaluated. Among the isolated ecdysonoids, Compounds **40**–**43** were the most potent. The results shown in Figure 7 suggest that ajugasterone C (Compound **42**) is equipotent to 2′-C-methylcytidine (positive control), with a better Seletive Index (SI). In addition, the most active ecdysone, Compound **42**, was also tested against other DENV serotypes. Ecdysone **42** was found to be active against all DENV serotypes, with the following EC_50_ values: DENV-1 (15.70 ± 2.36 μM), DENV-3 (9.48 ± 0.24 μM) and DENV-4 (12.15 ± 1.22 μM) with a high CC_50_ (the 50% cytotoxic concentration). After analyses of both the structure-activity relationship and molecular docking, the authors proposed that ecdysone **42** impairs DENV RNA replication by blocking the viral polymerase channel [54].

**Figure 8:**
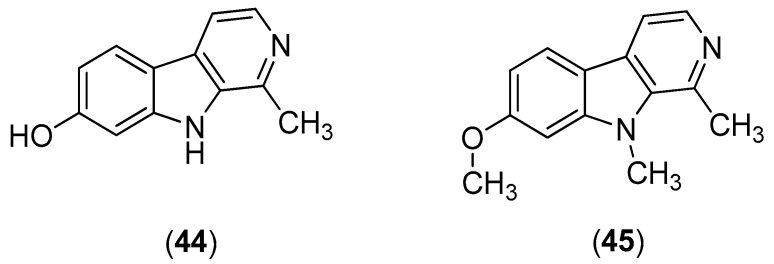
β-Carbolines harmol (Compound **44**) and its synthetic derivative 9-*N*-methylharmine (Compound **45**).

β-Carbolines are widespread plant and animal alkaloids that possess important biological activities [55]. Quintana and co-workers evaluated the effects of natural and synthetic β-carbolines; they found that the natural product harmol (Compound **44**) and its synthetic derivative 9-*N*-methylharmine (Compound **45**) were the most potent alkaloids, with inhibitory activities against DENV-2 and SI values of 56.2 and 61.3, respectively (Figure 8). Vero cells were infected with DENV at a multiplicity of infection (MOI) of 1 or 0.1 PFU/cell. At 48 h post-infection, the cells were lysed; and the supernatants were harvested; virus yields were quantified by plaque assay. The results indicate that the compounds likely act via viral enzyme inhibition. Harmol (Compound **44**) and 9-*N*-methylharmine (Compound **45**) were also found to possess inhibitory effects against DENV-1, DENV-3 and DENV-4, albeit with lower efficiencies than against DENV-2 [56].

### 3.6. In Silico Investigations of DENV Enzyme Inhibitors

**Figure 9:**
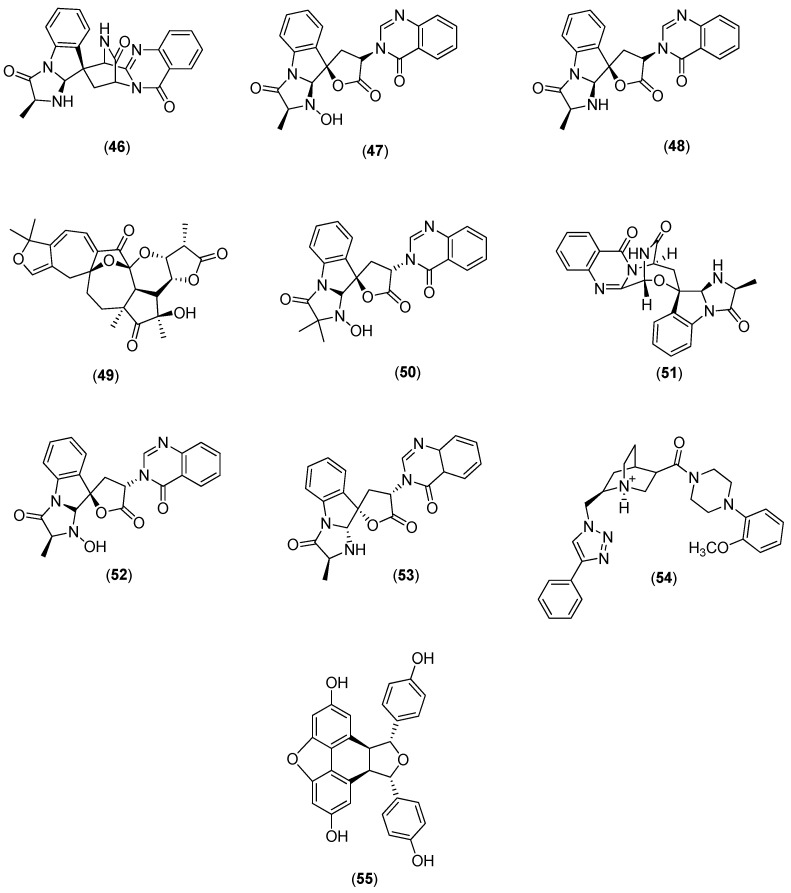
Structures of natural products **46**–**55** selected as leading candidate DENV RdRp inhibitors by Galiano and co-authors [57].

Galiano and co-authors conducted an in silico study to identify new inhibitors of the NS5 RdRp in the four DENV serotypes. They used a chemical library of 372,792 non-nucleotide compounds to perform molecular docking experiments at the binding site of the RNA template tunnel of the polymerase. After the screening process was completed, 39 compounds were identified as leading DENV RdRp inhibitor candidates. The selected compounds had a highly negative free energy variation (∆G) when docked to the binding site of the RNA template tunnel in the four DENV serotypes. In addition, the majority of the selected compounds had favorable druggability and optimal ADMET (absorption, distribution, metabolism, excretion, and toxicity) properties [57]. Among the 39 selected compounds, 10 (Compounds **46**–**55** in Figure 9) were natural products.

**Figure 10:**
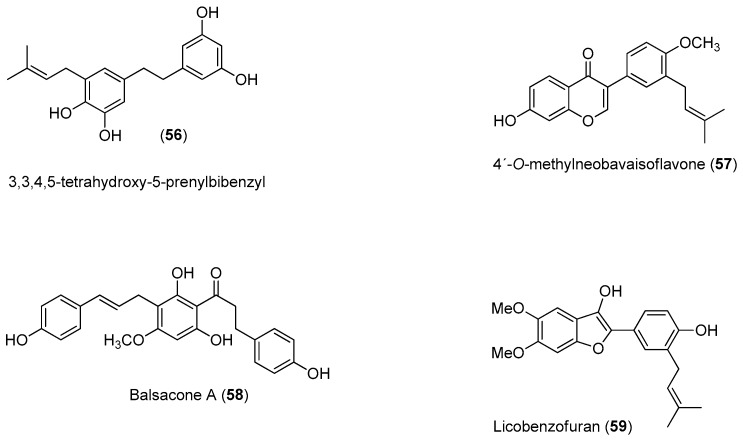
Identified compounds by Power and Setzer [58] as strongly docking ligands for DENV protein targets.

Recently, Power and Setzer reported an in silico investigation of natural products as potential antiviral agents against DENV protease (NS2B-NS3pro), helicase (NS3 helicase), MTase, RdRp and the virus envelope. A total of 2194 plant-derived natural products were docked. The compound set was composed of 290 alkaloids (68 indole alkaloids, 153 isoquinoline alkaloids, 5 quinoline alkaloids, 13 piperidine alkaloids, 14 steroidal alkaloids and 37 miscellaneous alkaloids), 678 terpenoids (47 monoterpenoids, 169 sesquiterpenoids, 265 diterpenoids, 81 steroids and 96 triterpenoids), 20 aurones, 81 chalcones, 349 flavonoids, 120 isoflavonoids, 74 lignans, 58 stilbenoids, 169 miscellaneous polyphenolic compounds, 100 coumarins, 28 xanthones, 67 quinones and 160 miscellaneous natural compounds. Polyphenolic compounds, flavonoids, chalcones and other phenolics were identified as the most strongly docking ligands for DENV protein targets [58], as shown in Figure 10 for selected ligands **56**–**59** for DENV NS2B-NS3.

## 4. Structural Biology of CHIKV

The genome of CHIKV is a positive-sense, single-stranded RNA (ssRNA) genome, consisting of two open reading frames: The 5′ end encodes nonstructural proteases necessary for the formation of viral replicase complexes, and the 3′ end encodes structural proteins necessary for receptor binding and cell membrane fusion [4]. Alphaviruses have a positive-sense ssRNA genome that is approximately 11 kb in length and encodes nine proteins. Four nonstructural proteins (nsP1-nsP4) are encoded by the 5′ end of the genome. The 3′ end of the genome encodes a polyprotein precursor containing three structural proteins: PE2 (the precursor of E3 and E2), E1 and the capsid protein (C) [59].

The nonstructural proteins nsP1, nsP2 and nsP4 possess enzymatic activities. They are involved in viral RNA genome synthesis, methylation of the 5′ cap of the viral genome and protein processing, among other activities [59].

### 4.1. nsP1

CHIKV nsP1 is a palmitoylated protein, consisting of 535 amino acids. The N-terminal region of nsP1 is responsible for the MTase and guanylyltransferase activities involved in capping and methylation of the newly-formed viral genomic and subgenomic RNAs. Recently, this protein was reported to play a crucial role in the downregulation of bone marrow stromal antigen-2 (BST-2), suggesting that nsP1 would be a potential target of BST-2-mediated therapeutics targeting CHIKV [7,59]. Elimination of nsP1 abolishes CHIKV replication. nsP1 genes are highly conserved in CHIKV strains and are important for virus proliferation in host cells. Therefore, nsP1 represents a rational target for antiviral therapies [60].

### 4.2. nsP2

The nonstructural protein nsP2 possesses numerous enzymatic activities and functional roles. This viral protein consists of an N-terminal domain with RNase and NTPase activities and a protease domain and an MTase-like region of unknown function at its C-terminus. In addition to its role as a cofactor of the viral polymerase complex, nsP2 is a virulence factor [4].

Similar to DENV NS3, CHIKV nsP2 cleaves viral polyproteins into four nonstructural proteins via the thiol protease complex at its C-terminus. The proteolytic activity of nsP2 plays an important role in the cleavage of nonstructural polyproteins, which are critical for viral replication. On the other hand, the RNA triphosphatase activity essential for RNA capping is found in the N-terminus of nsP2. The recently-solved crystal structure of CHIKV nsP2 might function as a crucial starting point for the development of novel antivirals targeting CHIKV [4,7,61,62]. The complete nucleotide sequence of the CHIKV genome revealed that nsP2 is the largest nonstructural protein: 798 amino acids long with a large, net positive charge [63].

### 4.3. nsP4

CHIKV nsP4 is an RdRp that is believed to participate in protein unfolding in the host cell, which helps replicate genomic RNA via a negative-strand RNA. CHIKV nsP4 also helps transcribe the 26S subgenomic mRNA, which encodes structural proteins [64,65].

Mature CHIKV nsP2 has been found to interact with both nsP1 and nsP4. Newly-synthesized simple strand RNAs may then be capped by nsP2 (RNA triphosphatase activity) and nsP1 (MTase and guanylyltransferase activities), suggesting that the interactions between nsP1, nsP2 and nsP4 are indispensable, as observed in a study of CHIKV nsPs [66,67]. Based on the crucial role of the CHIKV enzymes in viral replication, nsP1, nsP2 and nsP4 are important antiviral drug targets.

### 4.4. Direct Inhibitory Activities of Compounds against CHIKV Enzymes

**Figure 11:**
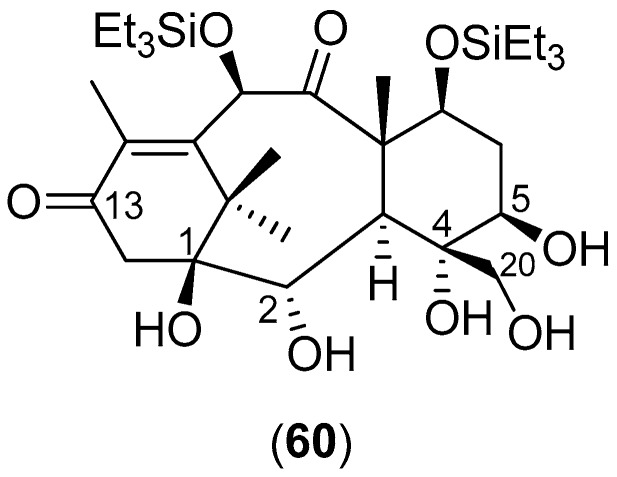
Structure of Compound **60**.

Lucas-Hourani and collaborators [8] conducted a phenotypic assay to identify CHIKV inhibitors that target nsP2 [8]. In their investigation, the inhibitory activities of 3040 compounds, at a final concentration of 10 μg/mL (average concentration of 30 ± 13 μM), were tested; the natural compound derivative **60**, shown in Figure 11, partially blocks nsP2 activity. This compound was then tested for its potential impact on CHIKV replication in vitro: HEK-293T cells were infected with CHIKV-Luc (MOI = 0.2) in the presence of the compound at 7.75, 15.5 or 31 μM (5, 10 or 20 μg/mL, respectively). A 50% reduction in *Renilla* luciferase activity was observed in the presence of Compound **60** at 31 μM (the IC_50_ value), thus demonstrating a weak, but statistically-significant inhibition of viral replication [8].

### 4.5. Indirect Inhibitory Activities of Compounds and Extracts against CHICKV Enzymes

The diterpenoids shown in Figure 3 were not evaluated against a specific CHIKV enzyme; however, all of them inhibited virus replication in the post-treatment assay of Vero cells, as seen in Table 1. Even though a specific target was not identified, the inhibitory effects suggest that enzymes involved in replication were affected. From the data presented in Table 1, the selective indices for Compounds **18**, **19** and **20** correspond to 23, 2.4 and 2.7, respectively. Compounds **18**–**20** were found to be more potent than chloroquine, which was used as a positive control [49].

Using a cell-based bioassay, the natural products shown in Figure 5 were also evaluated for inhibitory activities against CHIKV. Although Compounds **33**–**35** had antiviral effects (Table 2), no selectivity was observed. On the contrary, terpenes **36** (EC_50_ = 77 μM) and **37** (EC_50_ = 86 μM) presented lower, but selective activity against CHIKV (an SI of about three) [51].

**Figure 12:**
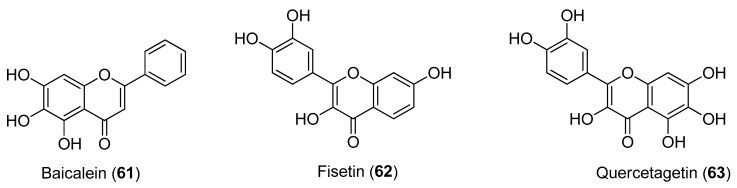
Structures of flavonoids **61**–**63**.

The flavonoids shown in Figure 12 were investigated for their inhibitory activities against CHIKV. qRT-PCR, immunofluorescence assay and western blot analyses all indicated that baicalein (Compound **61**), fisetin (Compound **62**) and quercetagetin (Compound **63**) affect CHIKV RNA production and viral protein expression, with IC_50_ values of 1.89 μg/mL (6.99 μM), 8.44 μg/mL (29.5 μM) and 13.85 μg/mL (43.52 μM), respectively, and minimal cytotoxicity. These data provide the first evidence of the intracellular anti-CHIKV activities of these compounds [68].

**Figure 13:**
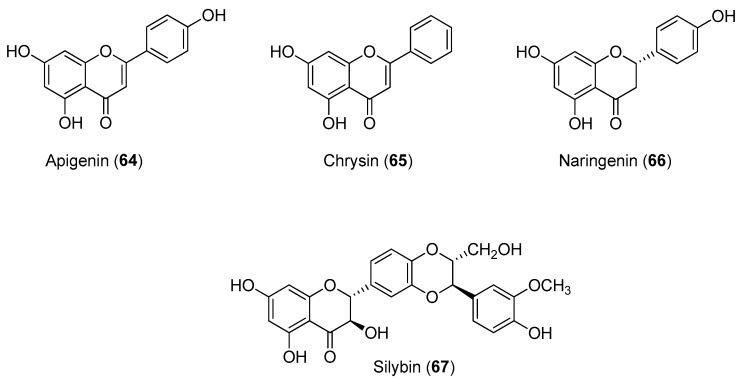
Structures of flavonoids **64** and **67**.

The CHIKV replicon cell line, BHK-CHIKV-NCT, was validated and used for screening 356 compounds, including 123 natural compounds and 233 clinically-approved drugs and other pharmaceutical compounds. After a 48-h exposure of the cell line to the compounds at a concentration of 50 mM, cellular EGFP appeared red, as the endpoint of the primary screen. The limit of the screen was set at a greater than 75% reduction of the EGFP signal, and the antiviral activities of all active compounds were confirmed in a second experiment that examined both EGFP and *Rluc* marker levels. The dose-dependent suppression of marker genes in the replicon vector after the 48-h exposure was observed for natural compounds apigenin (Compound **64**), chrysin (Compound **65**), naringenin (Compound **66**) and silybin (Compound **67**). The IC_50_ values for EGFP and *Rluc* are shown in Figure 13. All compounds showed low cytotoxicity with CC_50_ values greater than 200 μM, except for naringenin (Compound **85**), which had a CC_50_ value of 122.1 μM [69].

**Figure 14:**
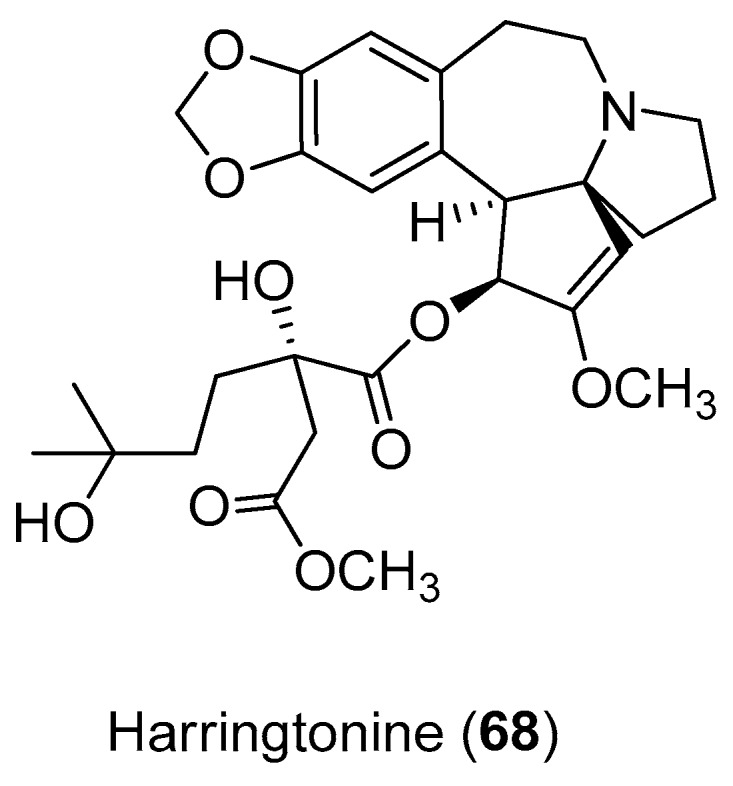
The alkaloid harringtonine (Compound **68**).

Harringtonine (Compound **68**) (Figure 14), a cephalotaxine alkaloid, potently inhibited CHIKV infection (EC_50_ = 0.24 μM), with minimal cytotoxicity (cell viability >90%). Harringtonine treatment resulted in a significant dose-dependent reduction in both negative- and positive-sense RNAs in both CHIKV strains, suggesting that it inhibited a phase in the CHIKV replication cycle that occurs before RNA production. Additionally, results also suggest that this alkaloid may inhibit CHIKV protein production, leading to a decrease in infectious virus titers [70].

Inhibitory activities of the aqueous, aqueous-ethanolic and ethanolic extracts of the roots of *Decalepis hamiltonii* and the leaves of *Vitex negundo* and *Hyptis suaveolens* were assessed against two strains (Asian and East Central South African lineages) of CHIKV in a post-treatment assay. Both the *V. negundo* ethanolic extract and the *H. suaveolens* aqueous-ethanolic extract were found to effectively inhibit the Asian strain, with both exhibiting 50% virus inhibition at a concentration of 15.62 μg/mL. The selectivity index of *H. suaveolens* (**16**) was higher than that of *V. negundo* (**2**) against the Asian CHIKV strain. No extracts were able to inhibit the East Central South African CHIKV strain, presumably because of the higher virulence and replicative features of the East Central South African strain compared to the Asian strain [71].

### 4.6. In Silico Investigations of CHIKV Enzyme Inhibitors

**Figure 15:**
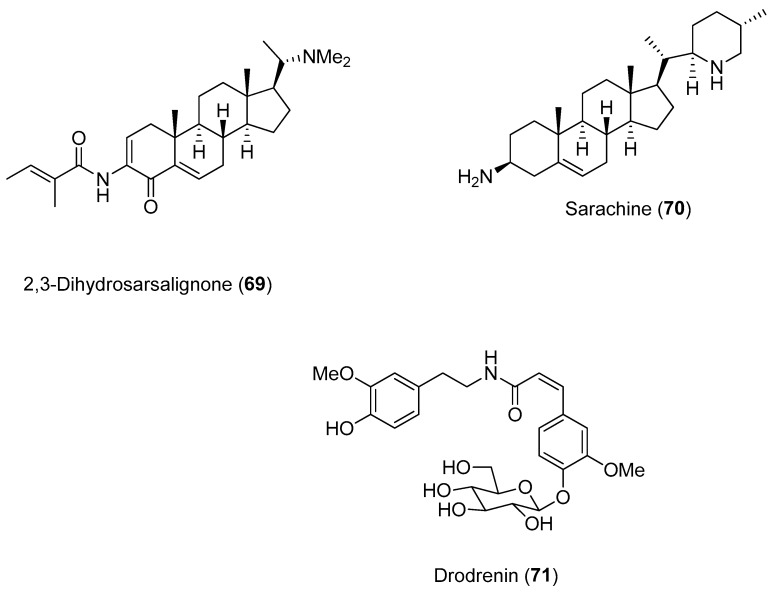
Structures of Compounds **69**–**71**, which were selective for the CHIKV nsP2 protease [72].

Setzer and collaborators conducted a molecular docking investigation to identify alphavirus protease inhibitors from natural compounds [72]. The inhibitory activities of 2174 natural substances were screened against several proteases, including nsP2 CHIKV. Several compounds presented notable docking energies and selectivities when screened against CHIKV nsP2, including the alkaloids 2,3-dehydrosarsalignone (Compound **69**) and sarachine (Compound **70**) (Figure 15). Both compounds occupy the same position in their lowest-energy docked position with the CHIKV protease.

The amide glucoside drodrenin (Compound **71**) was also selective for the CHIKV nsP2 protease; the glucose moiety of drodrenin occupies a position near the active site (close to amino acids Cys^1013^ and His^1083^).

## 5. Concluding Remarks

The exploitation of the natural product pool has afforded compounds and extracts that present important inhibitory effects on enzymes involved in DENV and CHIKV replication and protein expression. This is an important approach since inhibition of these enzymes can disrupt the virus life cycle. Most of the studies herein described were conducted in vitro using enzymatic assays [8,47,48,49,50,51,52] or viral strain/replicon on cells evaluating replication, inhibition and/or protein expression [49,51,52,53,54,56,68,69,70,71]. Three studies were conducted utilizing the in silico docking approach [57,58,72]. In vivo investigations of the described compounds and extracts were not reported. Most of the described enzymatic assays were carried out on DENV enzymes, and just one used CHIKV enzyme [8]. For CHIKV, assays performed on cells with a viral strain are one important option. Further investigations with heterologous expression of CHIKV enzymes for antiviral analyses are necessary so that a more profound evaluation of compounds against viral enzymes could be accomplished. In order to obtain a deeper understanding of the potential of the described natural products and extracts as antivirals for DENV and CHIKV, it is essential to carry out detailed in vitro and in vivo studies. It is important to mention that subsequent clinical trials are necessary, since efficiency in vitro is indicative, but it does not represent a guarantee of a good clinical trial. Considering the natural compounds described in this review, it is noticed that flavonoids are the ones that have been more extensively investigated.

Most of the investigations described in this review were performed with natural products from plant extracts. However, other sources of natural products, such as animals, microorganisms and marine organisms, should be explored towards the discovery of antivirals against DENV and CHIKV. It is also important to mention the fact that structures of natural compounds can be optimized, in terms of biological activities, via synthetic transformations. Thus, the compounds described in this review might be useful as starting points for synthetic endeavors towards the synthesis of more potent compounds against DENV and CHIKV. Finally, it is noticed from this review that, to date, there are few reports on the effect of natural products on dengue and chikungunya viral enzymes. In other words, the natural product pool is relatively unexplored in this regard. Considering the vast array of compounds available from nature, it is possible to anticipate that more investigations in this field will be described in the near future.

## Figures and Tables

**Table:** 

Compound	Polymerase Activity Inhibition ^a^	Cytotoxicity ^b^
**7**	Not active	0
**8**	14.8 ± 2.4	3.2
**9**	15.3 ± 1.4	0
**10**	9.0 ± 3.5	11
**11**	27.0 ± 6.5	0
**12**	7.4 ± 2.2	6
**13**	72.5 ± 15.3	0
**14**	4.2 ± 0.1	0
**15**	1.8 ± 1.2	0
**16**	2.9 ± 0.3	0
**17**	2.4 ± 0.3	0

^a^ IC_50_ (μM) values are the mean values ± SD (*n* = 3); ^b^ % of inhibition at 10 μg/mL.

**Table:** 

Compound	DENV RdRp Inhibitory Activity (IC_50_ in μM)
**18**	12.7 ± 0.2
**19**	3.1 ± 0.2
**20**	16.0 ± 1.3
3′-deoxy-GTP	0.02

**Table:** 

Compound	Polymerase Inhibition (IC_50_ in μM)
**21**	>50
**22**	9.3 ± 2.8
**23**	71.1 ± 1.2
**24**	23.8 ± 2.7
**25**	35.5 ± 3.8
**26**	24.3 ± 3.4
**27**	13.4 ± 1.9
**28**	39.8 ± 1.6
**29**	37.8 ± 3.6
**30**	9.5 ± 5.0
**31**	0.85 ± 0.10
3′-deoxy-GTP	0.02

**Table:** 

Compound	IC_50_ (in μM) DENV RdRp
**32**	2.5 ± 0.1
**33**	2.7 ± 0.4
**34**	2.2 ± 0.5
**35**	2.7 ± 0.4
**36**	>10
**37**	>10
**38**	>10
3′-deoxy-GTP	0.02

**Table:** 

Compound	EC_50_ (μM)	CC_50_ (μM)	SI (CC_50_/EC_50_)
**40**	19.61 ± 2.46	>700	>36.7
**41**	39.76 ± 2.14	>700	>17.6
**42**	10.05 ± 2.37	>700	>69.7
**43**	38.15 ± 2.40	>700	>18.3
2′-C-methylcytidine	11.20 ± 0.3	94.75 ± 4.15	>8.5

**Table:** 

Compound	CC_50_ (μM)	EC_50_ (μM)	SI
**44**	203.3 ± 1.2	3.3 ± 0.4	61.3
**45**	178.6 ± 11.5	3.2 ± 0.6	56.2

**Table:** 

Compound	EGPF (in μM)	Rluc (in μM)
**64**	22.5	28.3
**65**	46.8	50.2
**66**	25.8	30.0
**67**	71.1	59.8

**Table 1:** Antiviral activities of Compounds **18**–**20** against chikungunya virus (CHIKV) replication.

Compound	EC_50_ (in μM) CHIKV	CC_50_ (in μM) Vero Cells
**18**	1.5 ± 0.6	35 ± 8
**19**	1.9 ± 0.6	4.6 ± 0.8
**20**	3.9 ± 1.0	10.5 ± 0.1
Chloroquine	11.0 ± 2.1	100 ± 25

**Table 2:** Effects of Compounds **32**–**38** on CHIKV and Vero cells.

Compound	EC_50_ (in μM) CHIKV	CC_50_ (in μM) Vero Cells
**32**	>420	>420
**33**	>30	30
**34**	>23	23
**35**	>30	30
**36**	77 ± 26	>235
**37**	86 ± 9	>235
**38**	>426	>426
Chloroquine	11 ± 7	ND
